# Supplementary material for: Risk factors relate to the variability of health outcomes as well as the mean: A GAMLSS tutorial
Source: eLife. 2022 Jan 5;11:e72357. doi: 10.7554/eLife.72357 (PMC8791632; doi:10.7554/eLife.72357)
Supplement: Supplementary file 1. [file elife-72357-supp1.docx]

## Supplementary File 1a. Risk factors in relation to body mass index (BMI): differences in mean, variability and skewness estimated by GAMLSS

|  | | | NO Distribution Family | | BCCG Distribution Family | | |
| --- | --- | --- | --- | --- | --- | --- | --- |
|  | Risk Factor | % | Mean | SD | Median | CoV | Skewness |
| Father Class | I Professional | 6.8% | Ref | Ref | Ref | Ref | Ref |
|  | II Intermediate | 14.9% | 4.4 (1.1) | 19.4 (4.2) | 3.3 (1) | 11.1 (4.2) | -0.28 (0.28) |
|  | III Skilled Non-Manual | 14.7% | 4.9 (1) | 16.6 (4.2) | 4.3 (1) | 11 (4.2) | -0.02 (0.28) |
|  | III Skilled Manual | 46.2% | 7.3 (0.9) | 20.2 (3.7) | 6.8 (0.8) | 13.6 (3.7) | 0.12 (0.25) |
|  | IV Semi-Skilled | 12.6% | 8.9 (1.1) | 26.4 (4.3) | 8.7 (1) | 20.5 (4.3) | 0.43 (0.28) |
|  | V Unskilled | 4.9% | 9.4 (1.4) | 21.5 (5.4) | 9.7 (1.3) | 16.7 (5.4) | 0.59 (0.34) |
| Exercise | 0 days | 27% | Ref | Ref | Ref | Ref | Ref |
|  | 1 days | 11.9% | -2.6 (0.9) | -6.4 (3.2) | -2.6 (0.8) | -6.3 (3.2) | -0.18 (0.18) |
|  | 2 days | 15.1% | -2.5 (0.8) | -15.7 (2.9) | -2.1 (0.7) | -13.4 (2.9) | -0.2 (0.18) |
|  | 3 days | 15% | -3.6 (0.8) | -18.3 (2.9) | -3.1 (0.7) | -14.3 (2.9) | -0.11 (0.18) |
|  | 4 days | 8% | -4.2 (1) | -11.4 (3.7) | -4 (0.9) | -9 (3.7) | -0.14 (0.22) |
|  | 5 days | 9.5% | -3.4 (0.9) | -14.3 (3.4) | -3 (0.9) | -10.9 (3.4) | -0.08 (0.21) |
|  | 6-7 days | 13.4% | -3.8 (0.8) | -13.7 (3) | -3 (0.8) | -7.8 (3) | 0.17 (0.18) |

Estimates mutually adjusted for sex, social class and physical inactivity. Skewness is estimated as the Box-Cox power (that is, the power required to transform the outcome to a normally distribution); differences are the absolute difference in Box-Cox power in each subgroup estimated by GAMLSS. GAMLSS estimates multiple distribution moments simultaneously; thus, differences may not exactly correspond to descriptive comparisons reported above. NO: normal distribution family; BCCG: Box-Cox Cole and Green distribution family: SD: standard deviation; CoV: generalised coefficient of variation (SD/mean); GAMLSS: Generalized Additive Models for Location, Scale and Shape.

**Supplementary File 1b. Risk factors in relation to mental wellbeing (WEMWEBS): differences in mean, variability and skewness estimated by GAMLSS**

|  | | | NO Distribution Family | | BCCG Distribution Family | | |
| --- | --- | --- | --- | --- | --- | --- | --- |
|  | Risk Factor | % | Mean | SD | Median | CoV | Skewness |
| Father Class | I Professional | 6.2% | Ref | Ref | Ref | Ref | Ref |
|  | II Intermediate | 14.3% | -1.2 (0.9) | 2.5 (4) | -1.3 (0.8) | 4.7 (4) | -0.08 (0.29) |
|  | III Skilled Non-Manual | 14.3% | -2.2 (0.9) | 1.5 (4) | -2.2 (0.8) | 4.1 (4) | -0.02 (0.29) |
|  | III Skilled Manual | 46.5% | -3.6 (0.8) | 8.7 (3.6) | -3.6 (0.7) | 13.4 (3.6) | -0.21 (0.26) |
|  | IV Semi-Skilled | 13.6% | -5.8 (0.9) | 10 (4.1) | -6 (0.8) | 18.1 (4.1) | -0.38 (0.28) |
|  | V Unskilled | 5% | -6.2 (1.2) | 7.7 (5) | -6 (1) | 14.6 (5) | -0.11 (0.34) |
| Exercise | 0 days | 27.6% | Ref | Ref | Ref | Ref | Ref |
|  | 1 days | 11.9% | 3.1 (0.7) | -10.4 (2.9) | 2.9 (0.6) | -12.8 (2.9) | 0.01 (0.19) |
|  | 2 days | 14.7% | 5.7 (0.6) | -15.7 (2.7) | 5.5 (0.6) | -21.2 (2.7) | 0.16 (0.19) |
|  | 3 days | 14.9% | 6.5 (0.6) | -14.5 (2.7) | 6.6 (0.6) | -22.8 (2.7) | 0.42 (0.19) |
|  | 4 days | 7.9% | 7.7 (0.8) | -11.2 (3.4) | 7.1 (0.7) | -16.4 (3.4) | -0.26 (0.23) |
|  | 5 days | 9.6% | 5.2 (0.7) | -11.7 (3.1) | 5.1 (0.7) | -16.6 (3.1) | 0.08 (0.21) |
|  | 6-7 days | 13.6% | 4.3 (0.7) | -4.4 (2.8) | 4.3 (0.6) | -9.2 (2.8) | 0.11 (0.17) |

Estimates mutually adjusted for sex, social class and physical activity. Skewness estimates are the box-cox power in each subgroup (that is, the power required to transform the outcome to a normally distributed variable); differences are the absolute difference in box-cox power in each subgroup estimated by GAMLSS. NO: normal distribution family; BCCG: Box-Cox Cole and Green distribution family: SD: standard deviation; CoV: generalised coefficient of variation (SD/mean); GAMLSS: Generalized Additive Models for Location, Scale and Shape (GAMLSS).
